# NEIGHBORHOOD DISORDER MODERATES THE EFFECT OF SLEEP DIFFICULTIES ON COGNITIVE DECLINE AMONG OLDER AMERICANS

**DOI:** 10.1093/geroni/igad104.2639

**Published:** 2023-12-21

**Authors:** Darlingtina Esiaka, Elizabeth Luth

**Affiliations:** University of Kentucky, Lexington, Kentucky, United States; Rutgers University, New Brunswick, New Jersey, United States

## Abstract

Research suggests that the neighborhood where people live can be a risk or protective factor for various health outcomes, including cognitive decline. Similar to the impact of neighborhood on health outcomes, sleep quality has been linked to cognitive functions in older adults. However, limited studies have examined how neighborhood disorders moderate the effect of sleep on cognitive decline. Data were obtained from 2,494 respondents (1,065 men and 1,429 women) from wave 11 of National Health and Aging Trends (NHATS) data. Approximately 55% of the respondents were age 80+, and 74% identified as non-Hispanic White Americans. Sleep quality was operationalized as the absence of difficulties in falling and staying asleep. Neighborhood disorder (e.g., vandalism, graffiti) was based on observations by interviewers. Cognitive decline was operationalized as subjective reports of increasing or worse memory loss in the past 12 months. Moderated regression analysis using PROCESS macro (model 1) was performed to determine the associations between sleep, neighborhood, and cognitive decline. Results showed a significant main effect between neighborhood and cognitive decline (higher levels of neighborhood disorder result in an increase in cognitive decline, B= 0.369, p =.014), but no significant main effect between sleep and cognitive decline. There was a significant interaction between sleep and neighborhood on cognitive decline. Participants who reported higher-than-average sleep difficulties experienced a greater effect of neighborhood disorder on cognitive decline. Our findings could add to inform future health interventions and policy recommendations that can address modifiable sources of cognitive decline.

